# Supplementary material for: HIVprotI: an integrated web based platform for prediction and design of HIV proteins inhibitors
Source: J Cheminform. 2018 Mar 9;10:12. doi: 10.1186/s13321-018-0266-y (PMC5845081; doi:10.1186/s13321-018-0266-y)
Supplement: Supplementary file 2 — Additional file 2. Source code of HIVProtI web server. [file 13321_2018_266_MOESM2_ESM.zip › HIVprotI_Source-code/hivprotI/weka-3-6-6/doc/weka/associations/LabeledItemSet.html]

LabeledItemSet


---


|  |  |  |
| --- | --- | --- |
| **Overview** \| **Package** \| **Class** \| **Tree** \| **Deprecated** \| **Index** \| **Help** \| **Weka's home** | |  |
| **PREV CLASS**   **NEXT CLASS** | **FRAMES**    **NO FRAMES**     **All Classes** |  |
| SUMMARY: NESTED \| FIELD \| CONSTR \| METHOD | DETAIL: FIELD \| CONSTR \| METHOD |  |


---


## weka.associations Class LabeledItemSet

```
java.lang.Object
  weka.associations.ItemSet
      weka.associations.LabeledItemSet
```

**All Implemented Interfaces:**: java.io.Serializable, RevisionHandler

---

``` public class LabeledItemSet extends ItemSet implements java.io.Serializable, RevisionHandler ```

Class for storing a set of items together with a class label. Item sets are stored in a lexicographic
order, which is determined by the header information of the set of instances
used for generating the set of items. All methods in this class assume that
item sets are stored in lexicographic order.
The class provides the methods used for item sets in class association rule mining.
Because every item set knows its class label the training set can be splitted up virtually.

**Version:**
:   $Revision: 1.5 $

**Author:**
:   Stefan Mutter (mutter@cs.waikato.ac.nz)

**See Also:**: Serialized Form

---

**Constructor Summary**

`LabeledItemSet(int totalTrans,
int classLabel)`
  
          Constructor


| **Method Summary** | |
| --- | --- |
| `static FastVector` | `deleteItemSets(FastVector itemSets, int minSupport, int maxSupport)`             Deletes all item sets that don't have minimum support and have more than maximum support |
| `static Instances` | `divide(Instances instances, boolean invert)`             Splits the class attribute away. |
| `boolean` | `equalCondset(java.lang.Object itemSet)`             Compares two item sets |
| `boolean` | `equals(java.lang.Object itemSet)`             Tests if two item sets are equal. |
| `FastVector[]` | `generateRules(double minConfidence, boolean noPrune)`             Generates rules out of item sets |
| `static java.util.Hashtable` | `getHashtable(FastVector itemSets, int initialSize)`             Return a hashtable filled with the given item sets. |
| `java.lang.String` | `getRevision()`             Returns the revision string. |
| `static FastVector` | `mergeAllItemSets(FastVector itemSets, int size, int totalTrans)`             Merges all item sets in the set of (k-1)-item sets to create the (k)-item sets and updates the counters. |
| `static FastVector` | `pruneItemSets(FastVector toPrune, java.util.Hashtable kMinusOne)`             Prunes a set of (k)-item sets using the given (k-1)-item sets. |
| `static FastVector` | `singletons(Instances instancesNoClass, Instances classes)`             Converts the header info of the given set of instances into a set of item sets (singletons). |
| `int` | `support()`             Outputs the support for an item set. |
| `void` | `upDateCounter(Instance instanceNoClass, Instance instanceClass)`             Updates counter of item set with respect to given transaction. |
| `static void` | `upDateCounters(FastVector itemSets, Instances instancesNoClass, Instances instancesClass)`             Updates counter of a specific item set |

**Methods inherited from class weka.associations.ItemSet**

`containedBy, counter, hashCode, itemAt, items, pruneRules, setCounter, setItem, setItemAt, singletons, toString, upDateCounter, upDateCounters`

**Methods inherited from class java.lang.Object**

`getClass, notify, notifyAll, toString, wait, wait, wait`

**Constructor Detail**

### LabeledItemSet

```
public LabeledItemSet(int totalTrans,
                      int classLabel)
```

:   Constructor

    **Parameters:**: `totalTrans` - the total number of transactions: `classLabel` - the class lebel


**Method Detail**

### deleteItemSets

```
public static FastVector deleteItemSets(FastVector itemSets,
                                        int minSupport,
                                        int maxSupport)
```

:   Deletes all item sets that don't have minimum support and have more than maximum support

    :   **Parameters:**: `maxSupport` - the maximum support: `itemSets` - the set of item sets to be pruned: `minSupport` - the minimum number of transactions to be covered **Returns:**: the reduced set of item sets

---


### equals

```
public final boolean equals(java.lang.Object itemSet)
```

:   Tests if two item sets are equal.

    :   **Overrides:**: `equals` in class `ItemSet`
    :   **Parameters:**: `itemSet` - another item set **Returns:**: true if this item set contains the same items as the given one

---


### equalCondset

```
public final boolean equalCondset(java.lang.Object itemSet)
```

:   Compares two item sets

    :   **Parameters:**: `itemSet` - an item set **Returns:**: true if the the item sets are equal, false otherwise

---


### getHashtable

```
public static java.util.Hashtable getHashtable(FastVector itemSets,
                                               int initialSize)
```

:   Return a hashtable filled with the given item sets.

    :   **Parameters:**: `itemSets` - the set of item sets to be used for filling the hash table: `initialSize` - the initial size of the hashtable **Returns:**: the generated hashtable

---


### mergeAllItemSets

```
public static FastVector mergeAllItemSets(FastVector itemSets,
                                          int size,
                                          int totalTrans)
```

:   Merges all item sets in the set of (k-1)-item sets
    to create the (k)-item sets and updates the counters.

    :   **Parameters:**: `totalTrans` - the total number of transactions: `itemSets` - the set of (k-1)-item sets: `size` - the value of (k-1) **Returns:**: the generated (k)-item sets

---


### divide

```
public static Instances divide(Instances instances,
                               boolean invert)
                        throws java.lang.Exception
```

:   Splits the class attribute away. Depending on the invert flag, the instances without class attribute or only the class attribute of all instances is returned

    :   **Parameters:**: `instances` - the instances: `invert` - flag; if true only the class attribute remains, otherweise the class attribute is the only attribute that is deleted. **Returns:**: Instances without the class attribute or instances with only the class attribute **Throws:**: `java.lang.Exception` - exception if instances cannot be splitted

---


### singletons

```
public static FastVector singletons(Instances instancesNoClass,
                                    Instances classes)
                             throws java.lang.Exception
```

:   Converts the header info of the given set of instances into a set
    of item sets (singletons). The ordering of values in the header file
    determines the lexicographic order. Each item set knows its class label.

    :   **Parameters:**: `instancesNoClass` - instances without the class attribute: `classes` - the values of the class attribute sorted according to instances **Returns:**: a set of item sets, each containing a single item **Throws:**: `java.lang.Exception` - if singletons can't be generated successfully

---


### pruneItemSets

```
public static FastVector pruneItemSets(FastVector toPrune,
                                       java.util.Hashtable kMinusOne)
```

:   Prunes a set of (k)-item sets using the given (k-1)-item sets.

    :   **Parameters:**: `toPrune` - the set of (k)-item sets to be pruned: `kMinusOne` - the (k-1)-item sets to be used for pruning **Returns:**: the pruned set of item sets

---


### support

```
public final int support()
```

:   Outputs the support for an item set.

    :   **Overrides:**: `support` in class `ItemSet`
    :   **Returns:**: the support

---


### upDateCounter

```
public final void upDateCounter(Instance instanceNoClass,
                                Instance instanceClass)
```

:   Updates counter of item set with respect to given transaction.

    :   **Parameters:**: `instanceNoClass` - instances without the class attribute: `instanceClass` - the values of the class attribute sorted according to instances

---


### upDateCounters

```
public static void upDateCounters(FastVector itemSets,
                                  Instances instancesNoClass,
                                  Instances instancesClass)
```

:   Updates counter of a specific item set

    :   **Parameters:**: `itemSets` - an item sets: `instancesNoClass` - instances without the class attribute: `instancesClass` - the values of the class attribute sorted according to instances

---


### generateRules

```
public final FastVector[] generateRules(double minConfidence,
                                        boolean noPrune)
```

:   Generates rules out of item sets

    :   **Parameters:**: `minConfidence` - the minimum confidence: `noPrune` - flag indicating whether the rules are pruned accoridng to the minimum confidence value **Returns:**: a set of rules

---


### getRevision

```
public java.lang.String getRevision()
```

:   Returns the revision string.

    :   **Specified by:**: `getRevision` in interface `RevisionHandler` **Overrides:**: `getRevision` in class `ItemSet`
    :   **Returns:**: the revision


---


|  |  |  |
| --- | --- | --- |
| **Overview** \| **Package** \| **Class** \| **Tree** \| **Deprecated** \| **Index** \| **Help** \| **Weka's home** | |  |
| **PREV CLASS**   **NEXT CLASS** | **FRAMES**    **NO FRAMES**     **All Classes** |  |
| SUMMARY: NESTED \| FIELD \| CONSTR \| METHOD | DETAIL: FIELD \| CONSTR \| METHOD |  |


---
